# Synthesis, characterization and application of a novel polyazo dye as a universal acid–base indicator†

**DOI:** 10.1039/d2ra04930a

**Published:** 2022-10-03

**Authors:** Jannatul Naime, Muhammad Shamim Al Mamun, Mohamed Aly Saad Aly, Md Maniruzzaman, Md Mizanur Rahman Badal, Kaykobad Md Rezaul Karim

**Affiliations:** Chemistry Discipline, School of Science, Engineering and Technology, Khulna University Khulna-9208 Bangladesh s.mamun@ku.ac.bd; Department of Electrical and Computer Engineering at Georgia Tech Shenzhen Institute (GTSI), Tianjin University Shenzhen Guangdong 518052 China; Department of Chemistry, Khulna University of Engineering and Technology Khulna-9203 Bangladesh

## Abstract

A novel organic polyazo dye is synthesized by the diazotization of aromatic aniline, followed by coupling it with sulfanilic acid and *N*,*N*-dimethylaniline. Characterization was done by ^1^H-NMR, ^13^C-NMR, and FTIR spectroscopy. Differential scanning calorimetry (DSC) reveals that phase transition for this molecule is exothermic. The optical band gap is estimated from the absorption cutoff point using UV-Visible spectroscopy. Thermal gravimetric analysis (TGA) addresses the thermal stability of the molecule and is found to be at ∼250 °C. The structure of the synthesized molecule is analogous to that of methyl orange and contains three azo groups. These three azo groups help accept more than two protons and provide two p*K*_a_ values when diprotic acid or a mixture of acids is used in different titrations. Specifically, when a polybasic acid is in strong base titration, the p*K*_a_ values were found to be 3.5 and 9.1. Moreover, for strong base and (strong + weak) acid mixture titration, the p*K*_a_ values are found to be 9.2 and 3.3. Furthermore, the p*K*_a_ values are found to be 8.6 and 2.8 for (strong and weak) base mixture and (strong and weak) acid mixture titration, respectively. Owing to its increased proton accepting capacity, it can be found in the two pH ranges of 2.1–3.8 for orange color and 8.2–9.8 for yellow color, thus indicating a unique property as a universal indicator for acid–base titration. The dissociation constant of this dye is found to be 3.4 × 10^−6^, determined in a mixed aqueous solution of 10 wt% ethanol, and a linear relationship between p*K*_a_ and pH is observed in this solvent system.

## Abstract 2

A novel polyazo dye (SDMAPD3BS) was synthesized and its application as a universal acid–base indicator was investigated. This molecule can be used as a universal indicator for all types of acid–base reactions.

## Graphical abstract

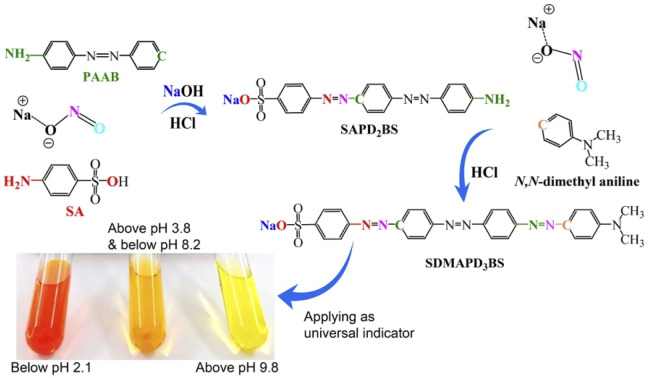

## Introduction

Owing to their broad spectrum of industrial applications (such as textiles, food coloring, pharmaceuticals, printing, and cosmetics), azo dyes are considered to be the most commonly used organic coloring materials.^1–5^ Azo compounds are water-soluble chemical synthetic compounds having a functional group with the general formula of A–N=N–B, where A and B are either aliphatic (alkyl, electron withdrawing) or aromatic (aryl, electron donating) functional groups, and (–N=N–) is a nitrogen–nitrogen double bound called azo group (chromophore).^6^ Their chemical structure contains a conjugated system, which is a chemical structure with alternating single and double bonds.^7^ Azo dyes exist in solid phase, most of which are salts, with the colored element usually being an anion mostly due to the presence of sulfonic acid groups; however, some cationic azo dyes exist.^8,9^ Azo dyes are prepared by diazotizing aromatic amines with electron-rich coupling agents (nucleophiles) containing one or more azo groups attached to aromatic molecules.^10^ Sulfur and nitrogen-containing azo dyes drew considerable attention owing to their attractive biological activities (anticancer, antioxidant, antifungal, antimicrobial, antiviral, and anti-inflammatory) endowing them with a wide range of pharmaceutical applications.^11–17^ It is noteworthy to mention that antibiotics containing azo dyes with the sulfonamide functional group were the first clinically effective chemotherapeutics used to systemically treat humans from bacterial infection.^18,19^ Moreover, azo dyes (such as methyl red and methyl orange) act as indicators in the acid–base and complexometric titrations of analytical chemistry by changing the color according to the extent of electron delocalization.^20–22^

Polyazo dyes contain more than two azo groups (–N=N–), thus increasing conjugation and enriching electron delocalization.^23^ It was reported that the more number of azo group the dye contains, the less the possibility of the dye degrading; for instance, single (mono-azo) or double (diazo) azo group continuing dyes are more likely to degrade than polyazo dyes.^24^ When the synthesized compound (azo dye) functions as an indicator, it changes color by withdrawing or releasing protons (or electrons) with the solution through a process called protonation. This change of color is more stable when the number of azo group (–N=N–) increases.^25^ Therefore, polyazo dyes can be more stable and be used as an indicator. Ideally, lower delocalization changes the absorption from longer to shorter wavelengths (hypsochromic shift), while more delocalization moves the absorption from shorter to longer wavelengths (bathochromic shift), thus making the reflected light more red.^26^ According to this shifting, the stability of polyazo dyes can be characterized by the impact of acid and base on the visible absorption maxima by analyzing the band gap (HOMO and LUMO) using cyclic voltammetry.^27^ Furthermore, polyazo dyes constitute a significant category of organic dyes used in a variety of possible practical applications.^19,28–33^

Acid–base indicators are organic dyes displaying different colors in solutions with the change in the pH levels. The chemical structure of azo dyes containing phenol rings with the azo group along with thiol as functional groups leads to high conjugation and unique mechanism of changing colors in titration.^34^ Furthermore, owing to their simple synthesis, stability, high efficiency, and lower degradability in titration, azo dyes have been extensively utilized as acid–base indicators during the last decade.^35–41^ These indicators are mostly preferable for detecting the end point of strong acid–weak base titrations. Also, they are used occasionally in the detection of the end point of strong acid–strong base titrations. Due to the limitation of their active pH ranges, they are not used in strong base–weak acid and weak acid–weak base titrations. For example, methyl orange is not suitable for strong base–weak acid titrations. When a titration between a weak species and strong species is conducted, the indicator used must have a pH range in the side of the strong species. In case of strong base (NaOH) and weak acid (CH_3_COOH) titration, the indicator used must have a pH range in the basic region but methyl orange has a pH range in the acidic region. Similar limitation was reported for other dye indicators such as bromocresol green and chlorophenol red by Stancil Cooper.^42^ To overcome the above-stated limitations, he reported about the mixed indicator bromocresol green and methyl red for bicarbonate titration. However, using mixed indicators is expensive and the choice of mixed indicators is a tedious job, which highlights the importance of a universal indicator. Again, the optimum suitability of a double or mixed indicator will depend not only on the properties of individual indicators mixed but also on their relative proportions chosen for simultaneous use.^43^ To the best of our knowledge, there is no reported dye that can detect the end point of polybasic acid–polyacidic base titrations; thus, a mixed indicator is used to overcome these limitations.^42,44^

Herein, we synthesized a novel polyazo dye and investigated its application as a universal acid–base indicator. The novel compound synthesized in this study can be used in strong acid–strong base, weak acid–weak base, and polybasic acid–polyacidic base titrations due to its unique double pH ranges and p*K*_a_ values. Characterization was validated by FTIR spectroscopy, ^1^H NMR, and ^13^C NMR. Furthermore, thermal gravimetric analysis (TGA) was performed to investigate the thermal stability of the molecule. Differential scanning calorimetry (DSC) revealed that phase transition for the synthesized molecule is exothermic. Cyclic voltammetry (CV) measurements were conducted to evaluate the band gap of the synthesized molecule. To verify the applicability of the synthesized polyazo dye as an indicator, it was tested at different acid–base conditions. Experiments (availability of π-delocalized electrons and stability of the polyazo compound) revealed that the synthesized molecule can be used as a universal indicator for all types of acid–base reactions, thus signifying the benefit of having two p*K*_a_ values.

## Experimental section

### Materials and methods

All starting materials and reagents were purchased commercially: aniline (C_6_H_5_NH_2_), hydrochloric acid (HCl), and sodium hydroxide (NaOH) were purchased from E. Merck (Germany), and sodium nitrite (NaNO_2_) and sodium chloride (NaCl) were purchased from Sigma-Aldrich (Germany) and used as received unless specified. The intermediates *p*-aminoazobenzene (PAAB) and sodium diazenyl benzene sulfonate (SAPD_2_BS) were prepared according to previously reported procedures, as shown in Schemes S1 and S2, respectively, presented in the ESI.†^45^ An FT-IR spectrophotometer (SHIMADZU 4200, Kyoto, Japan) was used for recording the IR-spectra of the synthesized molecule. A BRUKER 400 spectrometer (BRUKER, MA, USA) was used for recording the ^1^H-NMR spectra of the synthesized dyes. MeOD was used as the solvent and TMS was utilized as the internal standard on a Varian 500 MHz spectrometer AV-500 at 25 °C. Elementary analysis was carried out using an Elementary analyzer (Vario MICRO cube), made in Germany.

The absorption spectra of the compounds were measured by a UV-Vis double-beam spectrophotometer (LABOMED, Inc., UVD-3200 PC, CA, USA) at 298 K through a quartz cell with a path length of 1 cm. The compound was dissolved in dichloromethane to make a solution of concentration 1 mM and the optical band gap (*E*_gap,optical_) was obtained by the following eqn (1) from the onset wavelength of the UV-Visible spectra.^46^

1 *E*_gap,optical_ = *h* × *c*/*λ*_offset_ = 1240/*λ*_offset_

where *λ*_offset_ (nm) represents the absorption edge wavelength.

The synthesized compound has potential to be used in few applications that require thermal stability at high temperatures such as polymer-based high temperature processed food products, Kjeldahl method, in which the mixed indicator is used to determine the total nitrogen content of any food product, the amount of iron in samples (food, liquid, and chemical) using KMnO_4_ and oxalic acid, and thermodynamics of neptunium(v) complexation with sulfate in aqueous solutions in ground water treatments. Therefore, the thermal degradation (TGA and DSC) of the synthesized compound was carried out on a NETZSCH STA 449F3 (NETZSCH Group, Selb, Germany) instrument under purified nitrogen gas flow at a heating rate of 10 °C min^−1^. The CV was carried out on a μStat 400 Drop Sens (Metrohm DropSens, Asturias, Spain) microcomputer-based electrochemical analyzer at room temperature. In the CV analysis, a conventional three-electrode configuration employing a glassy carbon electrode as the working electrode, an Ag/AgCl electrode as the reference electrode, and a Pt wire as the counter electrode. Redox potential was measured in a 0.1 M Na_2_SO_4_ aqueous solution at a scan rate of 50 mV s^−1^ taking the concentration of our molecule as 3.1 × 10^−5^ M. The experimental electronic band gap (*E*_gap,electronic_) was estimated using eqn (2).^46–48^ HOMO and LUMO were estimated from the onset oxidation and reduction potential, respectively.

2 *E*_gap,electronic_ = *E*_HOMO_ − *E*_LUMO_

#### Method development

Sodium 4-((*E*)-(4-((*E*)-(4-((*E*)-(4-(dimethylamino)phenyl)diazenyl)phenyl)diazenyl)phenyl)diazenyl)benzenesulfonate (SDMAPD_3_BS).^49,50^

**Scheme 1:**
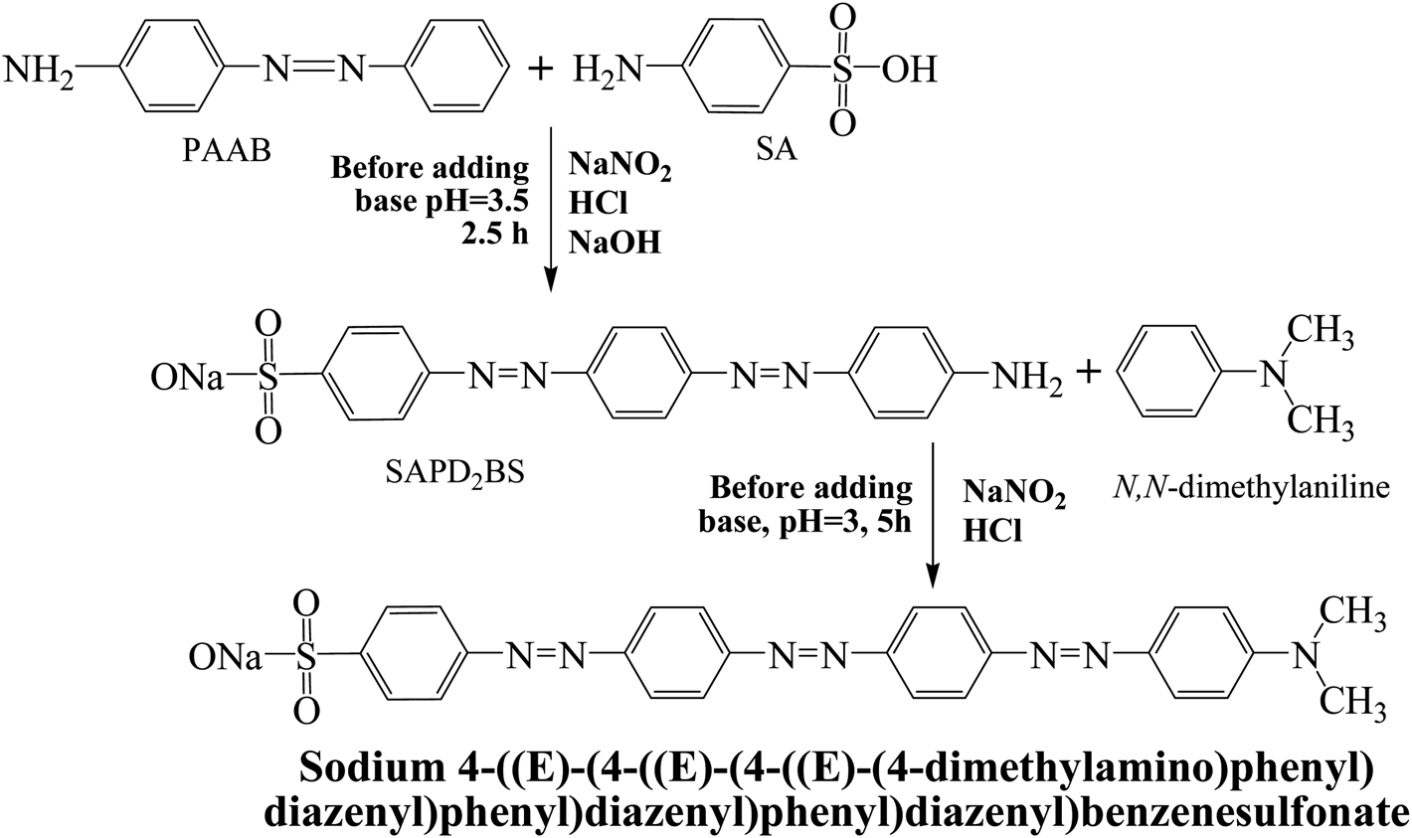
Synthesis routes of sodium 4-((*E*)-(4-((*E*)-(4-((*E*)-(4-(dimethylamino)phenyl)diazenyl)phenyl)diazenyl)phenyl)diazenyl)benzenesulfonate (SDMAPD_3_BS).

A solution of SAPD_2_BS (3.8 g, 10 mmol), sodium carbonate (0.6 g, 5.6 mmol), and 25 mL of water was first mixed in a round bottom flask. Following, the flask was placed in a hot (90 °C) water bath until a clear solution was obtained. Next, sodium nitrite (2 g, 29 mmol, 98%) was added to the mixture and then the mixture containing flask was placed in an ice water bath until a significant amount of solid was precipitated. Then, the above mixture was added into a basic solution of *N*,*N*-dimethylaniline (1.8 g, 17 mmol) and then the flask was placed in an ice-water bath for 25–30 minutes. Next, the mixture was heated until the boiling commenced, and then, sodium chloride (3 g) was added until it dissolved. Next, the stirring was stopped and the mixture-containing flask was left to slowly cool down in an ice water bath for 80–90 min. After drying, the crude product was recrystallized with *n*-hexane and DCM, as shown in Scheme 1. Maroon powder, mp 226 °C, yield 81%. The IR (KBr) spectrum of the product (*ν* cm^−1^): 3050, 1600, 1475, 1450, 1400, 1380, 1270, 1180, 840, 820, 680. ^1^H NMR (400 MHz, MeOD): *δ* = 8.06 (d, *J* = 8.8 Hz, 2H, Ar), 7.92 (d, *J* = 8.8 Hz, 2H, Ar), 7.57 (t, *J* = 1.6 Hz, 4H, Ar), 7.57 (d, *J* = 7.6 Hz, 2H, Ar), 7.55 (t, *J* = 1.6 Hz, 4H, Ar), 7.52 (d, *J* = 7.2 Hz, 2H, Ar), 4.88 (s, 6H, 2CH_3_), and 3.33 (MeOD) is the solvent peak. ^13^C NMR (MeOD): *δ* = 130.762; 129.099; 127.583; 126.342; 126.062; 125.768; 122.635; 121.860 (over lapping peaks); 121.721; 121.639. Elemental analysis found: C, 50.89; H, 4.62; N, 13.83, S, 6.49%.

Density functional theory (DFT) was analyzed using Becke's three-parameter hybrid functional combined with B3LYP functional and the non-local correlation functional of LYP.^51^ The B3LYP/6-311G +(d,p) and APFD/6-311G +(d,p) basis set was used to optimize the synthesized structures. In order to find the local minima for stationary points, harmonic vibrational frequencies were performed at the same level of theory. All calculations were obtained using Gaussian 16, revision C.01 series of programs.

## Results and discussion

### Synthesis, optical absorption, electrochemical and thermal property

Herein, we have synthesized a modern dye containing polyazo groups. All benzene rings, both of the left and right side of the azo bond (–N=N–) are arranged in such a way that minimum steric hindrance is present in the structure. Therefore, the most stable configuration is zigzag shaped having E (entgegen) configuration in which the benzene rings remain on opposite sides of the azo group. A synthesis route of SDMAPD_3_BS is depicted in Scheme 1 where a flat structure is shown.

**Fig. 1:**
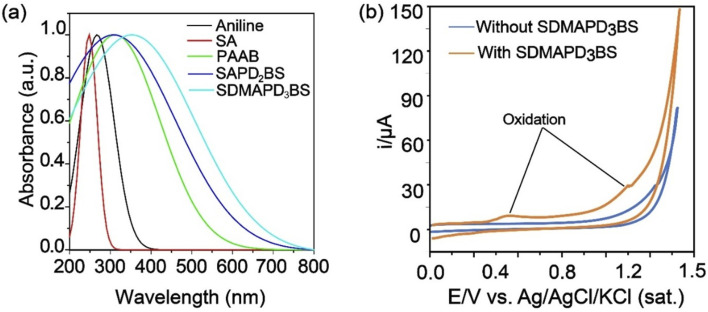
UV-Visible spectra of aniline, SA, PAAB, SAPD_2_BS, and SDMAPD_3_BS (a). Red shifting occurred by the continuous addition of the starting material. Cyclic voltammograms with and without SDMAPD_3_BS at a scan rate of 50 V s^−1^ (b).

The absorption spectra of the starting materials such as aniline, sulfanilic acid (SA), and the products of different stages, namely PAAB, SAPD_2_BS, and SDMAPD_3_BS were recorded in the range of 200–800 nm, as shown in Fig. 1a. By looking at the figure, one can observe that the peaks shifted from left to right (bathochromic shift) with the addition of the reactant due to the increase in the number of azo groups. Because of red shifting, the diluted ethanolic solution of SAPD_2_BS was absorbed at 306 nm with a significant peak and the highest coefficient was of 3.49 × 10^6^ M^−1^ cm^−1^. Subsequently, azo groups were increased by adding *N*,*N*-dimethyl aniline in the terminal position and the SDMAPD_3_BS synthesized solution was presented at a bathochromic peak at 374 nm with a higher maximal coefficient of 5.54 × 10^6^ M^−1^ cm^−1^.^52^ It is noteworthy to mention that the absorption coefficient and the band gap for the synthesized compound increased and decreased, respectively, by adding *N*,*N*-dimethyl aniline to modify the end unit of the synthesized polyazo dye. In addition, the optical band gap of SDMAPD_3_BS was estimated using eqn (1) to be 1.89 eV.

The electrochemical performance of SDMAPD_3_BS was studied by CV using a glassy carbon working electrode in a supporting electrolyte of 0.1 M Na_2_SO_4_ aqueous solution under nitrogen atmosphere. The first oxidation peak of SDMAPD_3_BS in the cyclic voltammogram corresponded to the oxidation of the azo compound where the azo group (–N=N–) was converted to (=N^+^=N) to produce N^0/I^ at a potential of 0.43 V *vs.* Ag/AgCl, as shown in Fig. 1b. Furthermore, the second oxidation peak corresponded to the second oxidation at a potential of 1.11 V *vs.* Ag/AgCl (for two numbers of N^0/I^) where two azo groups (–N=N–) in one molecule of SDMAPD_3_BS were converted to (=N^+^=N^−^). In the reduction process, there was no significant current change observed indicating that the reduction process was absent in SDMAPD_3_BS.

Furthermore, the onset oxidation and reduction potentials of the synthesized compound were used to calculate the energy levels of the highest occupied molecular orbital (HOMO) and lowest unoccupied molecular orbital (LUMO) and were found to be −4.83 eV and −2.94 eV respectively, as shown in Fig. 1b. The electrochemical band gap of the final compound was estimated using eqn (2) to be 1.85 eV, and it is consistent with the value of the optical band gap. Furthermore, the thermal property of SDMAPD_3_BS was examined using TGA and DSC, confirming it was an exothermic reaction and soluble in a common organic solvent with good thermal stability up to 250 °C under N_2_ atmosphere. It is noteworthy to mention that the compound annihilation started from 185 °C up to complete obliteration, as shown through the TGA and DSC curves of Fig. S1 in the ESI.†

### SDMAPD_3_BS as acid–base indicator

**Fig. 2:**
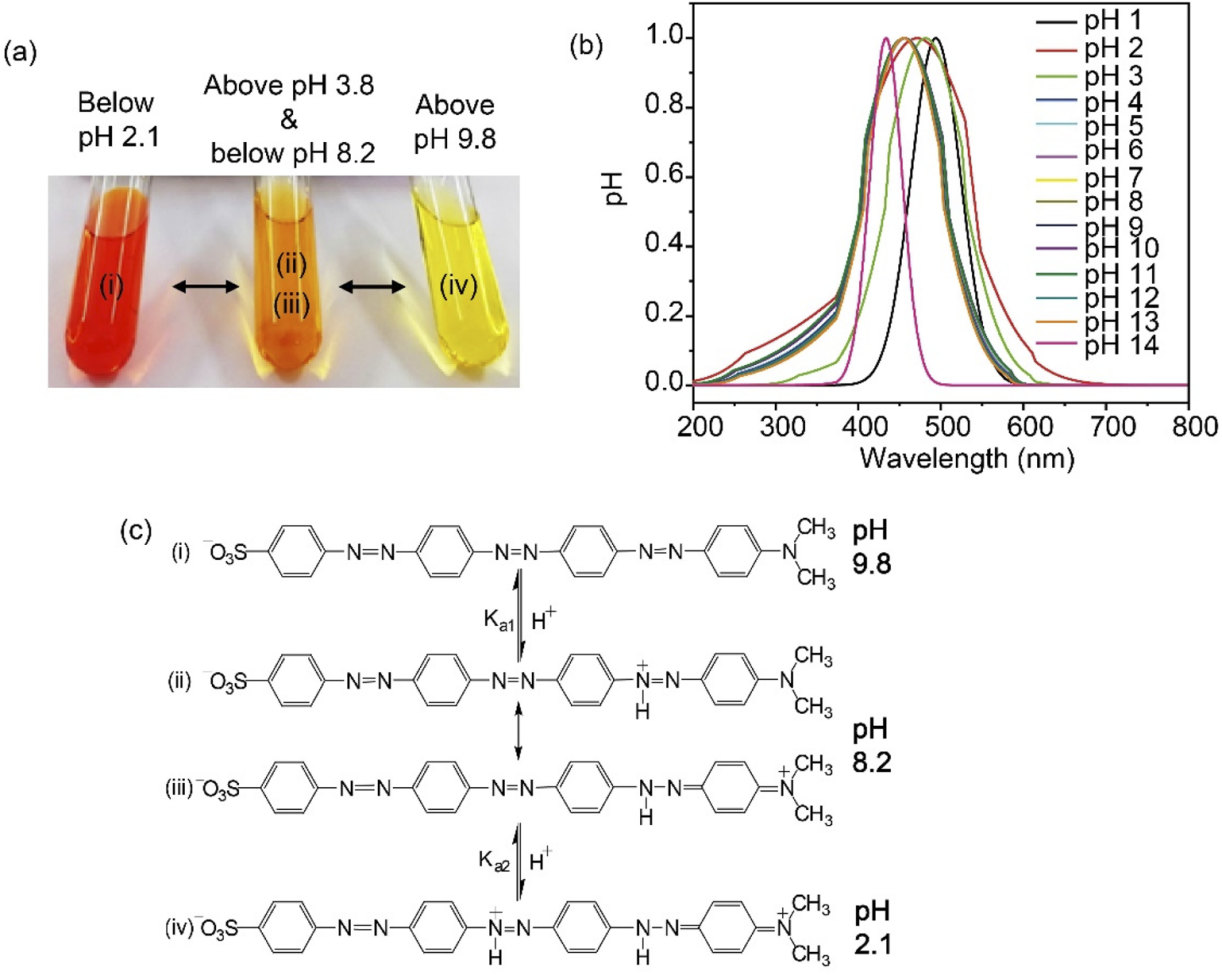
(a) The significant colors with corresponding pH ranges; (b) the normalized UV-visible spectra of SDMAP_3_DBS solutions at the pH range of 1–14; (c) the proposed structures according to the changing of pH levels (c); that is (i) alkaline form of SDMAPD_3_BS, (ii) the monoprotonated zwitterionic structures under acidic conditions, (iii) the resonant form of azonium, and (iv) the bis-protonated cationic quinoid structures under acidic conditions.

SDMAPD_3_BS displayed three significant major colors, namely red, orange, and yellow in all pH ranges, exhibiting visual hypsochromic shift with the increase in the pH level in the solution. This phenomenon was observed by following the spectra of peaks using a UV-visible spectrophotometer of the synthesized azo dye in a solution at three different pH levels (1, 7, and 14), as shown in Fig. 2a. This visual blue shift was confirmed by a peak shift, indicating that the SDMAPD_3_BS structure lost conjugation with the increase in the pH level.^27^ This observation can also explain the mechanism of change in the color of SDMAPD_3_BS as an indicator in acid–base titrations.

The hypsochromic shift of SDMAPD_3_BS occurred due to the two protons withdrawn by the azo group from the acidic solution in two steps, and thus converted into a source of proton, as shown in Fig. 2b. Therefore, it exposed its acidic nature in solution and formed an ionic structure gradually. The zwitterionic and cationic quinoid structures of SDMAPD_3_BS indicate charge separation in the ground state, as represented in Fig. 2c(ii)–(iv). Owing to zwitterion formation, the structures shown in Fig. 2c(ii) and (iii) are more stable than the structures displayed in Fig. 2c(i) and (iv). Moreover, the conjugation system was destroyed because of the formation of the quinoid structure; thus, the structure presented in Fig. 2c(iv) is more stable than Fig. 2c(i).^53–55^

Furthermore, this behavior of SDMAPD_3_BS was confirmed by determining the dissociation constant, showing two different dissociation constants at two different p*K*_a_ values as further explained in ESI.† The dissociation constant of SDMAPD_3_BS was 1.16 × 10^−3^ and 1.65 × 10^−9^ at p*K*_a_ values of (2.94 ± 0.01) and (8.78 ± 0.02), respectively, in a 10 wt% EtOH–H_2_O mixed solution.^56^ When SDMAPD_3_BS accepted one proton, the rest of the compound conjugated (aromaticity) and then converted into an ionic form in the second phase similarly. For that reason, the *K*^1^_d_ of SDMAPD_3_BS was lower than *K*^2^_d_ with a linear relationship between p*K*_a_ and pH (as shown in Fig. S2†) for an average value of *K*_d_ and p*K*_a_ at 3.4 × 10^−6^ and 5.47 ± 0.01, respectively. Finally, SDMAPD_3_BS dissociated at low pH levels.

Furthermore, adding more azo groups affected the solubility of the compound in polarity basis and increased the stability of the compound.^35^ By changing its structure at different pH levels of the solution, it performed as an indicator. Furthermore, the synthesized compound (SDMAPD_3_BS) exhibited two equivalent points especially for polyacidic/polybasic titration due to their pH ranges; one pH range belongs to a more acidic area and other to a less basic area. There were seven types of acid–base titrations done, where the color of the solution changed dramatically with the addition of 1–2 drops of SDMAPD_3_BS. The endpoints were recorded for all mentioned titrations and presented in Table 1.

**Table 1:** Titration of acid (0.1 M) against base (0.1 M)

Acid	Base	Equivalence point	
		Experimental	Reference^42,57,58^
HCl	NaOH	7.05	7.0
CH_3_COOH	NaOH	8.4	8.73
HCl	NH_4_OH	4.55	5.27
CH_3_COOH	NH_4_OH	7.2	∼7.0
H_2_SO_4_	NaOH	4.9 & 10.0	7.14
H_3_BO_3_ & HCl	NaOH	4.75 & 9.90	a
H_3_BO_3_ & HCl	NH_4_OH & NaOH	3.0 & 8.35	a

a No reference value has been found.

**Fig. 3:**
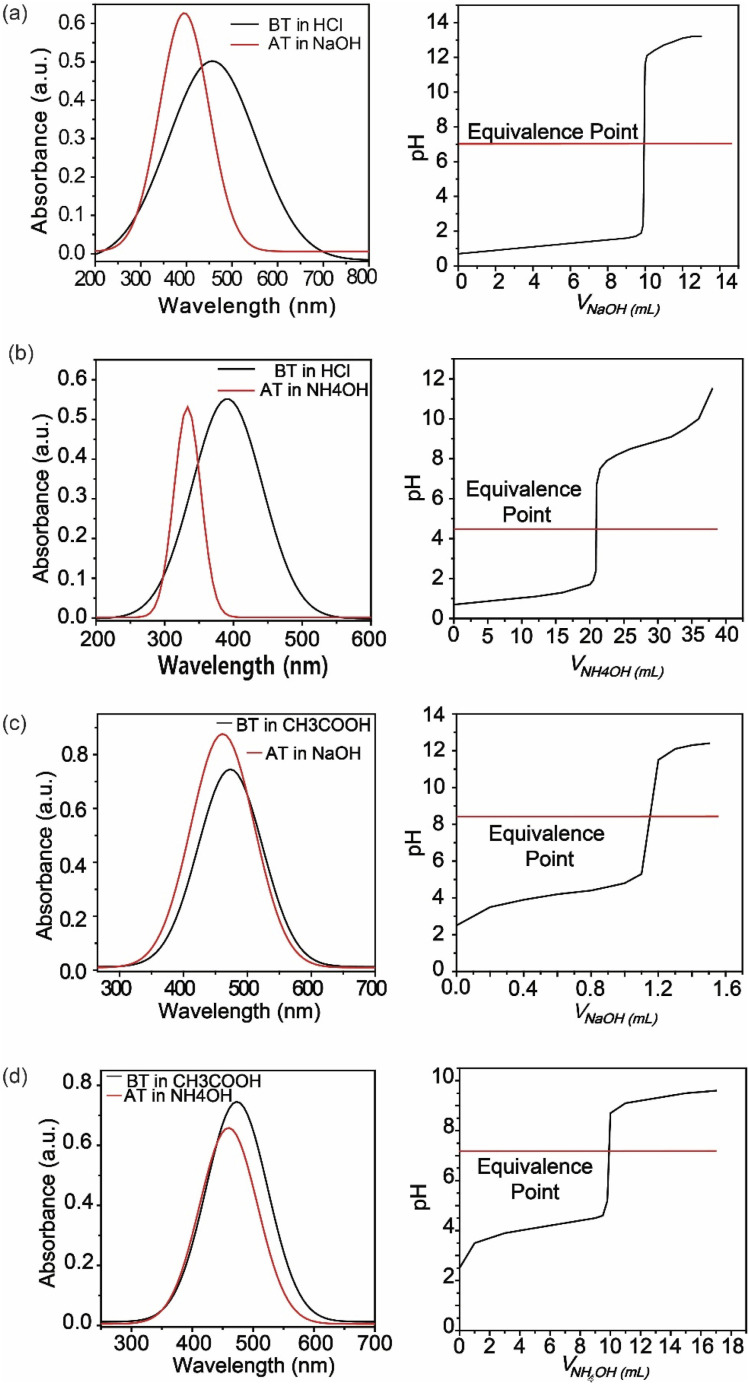
UV-Visible spectra of the acidic solution was shifted from right to left during titration and titration between strong base (SB) and strong acid (SA) (a), strong base and weak acid (WA) (b), strong acid (SA) and weak base (WB) (c), and weak acid and weak base (d). *BT = before titration; *AT = after titration; *MT = middle-state titration.

**Fig. 4:**
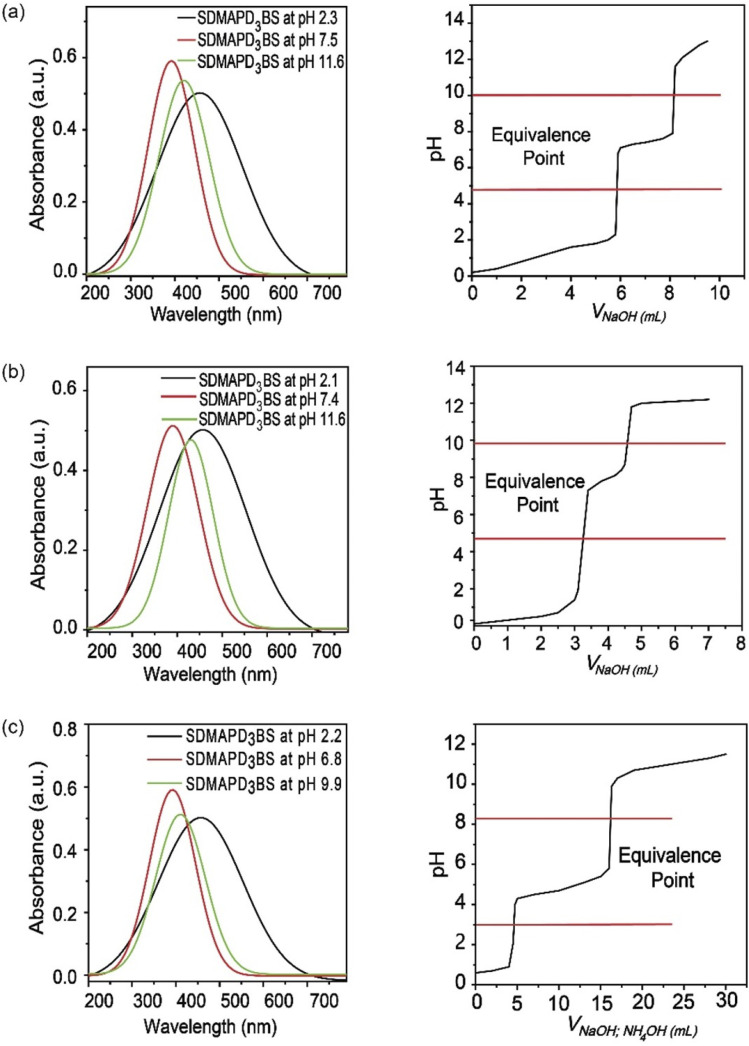
UV-Visible spectra of the acidic solution shifted from right to left during titration and titration between polybasic acid (PA) and strong base (a), strong acid and weak acid and strong base (b), strong acid–weak acid and strong base–weak base (c).

Since SDMAPD_3_BS has two pH ranges and three azo groups, it can be assumed that it shows one pH range of 8.2–9.8 at a wavelength of 387–393 nm with yellow color when it takes one proton from the solution in the first step to convert itself from basic to acidic nature. Furthermore, it shows a pH range of 2.1–3.8 at a wavelength of 454–458 nm with orange color when it takes two protons from the solution, as shown in Fig. 3(a)–(d). Furthermore, in the case of polybasic acid–base titrations, the participating compounds containing more than one ionizable ion per molecule require more electron or proton accepting nature-based indicators that can show color change at their equivalence point and endpoint. Since the synthesized compound contains two pH ranges with two p*K*_a_ values, thus helping to determine the accurate endpoint of polybasic acid and polyacidic base titrations, at which two equivalence points were found, as can be observed in Fig. 4(a) and (b). Moreover, owing to the presence of more than one ionizable ion, the synthesized compound was able to determine the endpoint of titrations containing a mixture of acids and bases, as shown in Fig. 4 (c).

**Fig. 5:**
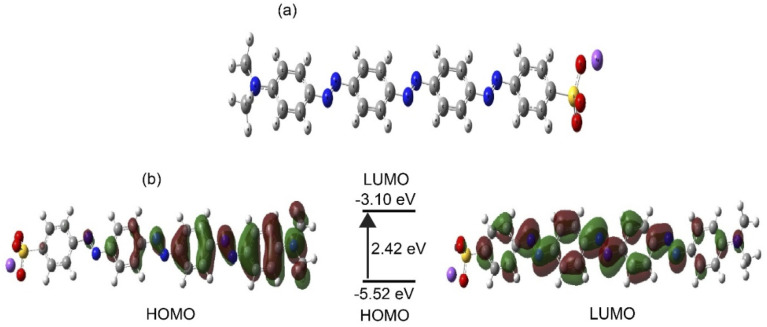
The optimized 3D structure of SDMAPD_3_BS (a). Frontier molecular orbital distributions and energy levels of SDMAPD_3_BS by DFT (b).

The optimized chemical structure of the synthesized compound (SDMAPD_3_BS) is shown in Fig. 5a. The frontier molecular orbital distributions and energy levels of SDMAPD_3_BS obtained by the DFT computational method are shown in Fig. 5b. The energy difference between the HOMO (−5.52 eV) and LUMO (−3.10 eV) was found to be 2.42 eV by DFT calculation. It is noteworthy to mention that this energy difference is larger than the band gaps of 1.89 eV and 1.85 eV, obtained from optical band gap, and the onset oxidation and reduction potential, respectively. Band gap was calculated by DFT in the gas phase where only intrinsic properties of the molecules were exhibited. On the other hand, the band gap was measured experimentally in a solid state on a glass in air in which the band bending may occur due to an external molecule. Therefore, the band gap value from DFT was slightly higher than the experimental value, which is consistent with the previous report.^59^

## Conclusion

Herein, a novel polyazo dye was synthesized and its application as a universal acid–base indicator was investigated. Owing to its unique double pH ranges and p*K*_a_ values, the synthesized compound can be used in strong acid–strong base, weak acid–weak base, and polybasic acid–polyacidic base titrations. To verify the applicability of the synthesized compound as an indicator, different acid–base titrations were done. Interestingly, this molecule can be used as a universal indicator for all types of acid–base reactions, which signifies the benefit of having two p*K*_a_ values.

## Author contributions

Jannatul Naime: conduct experiment and paper writing; Muhammad Shamim Al Mamun: research idea, supervision, and review manuscript; Mohamed Aly Saad Aly: manuscript writing and review manuscript; Md. Maniruzzaman: DFT and review manuscript; Md Mizanur Rahman Badal: DFT; Kaykobad Md Rezaul Karim: review manuscript.

## Conflicts of interest

Authors declare no conflict of interest.

## Supplementary Material

RA-012-D2RA04930A-s001

## References

[cit1] Benkhaya S., M'rabet S., El Harfi A. (2020). Heliyon.

[cit2] Gürses A. , Açıkyıldız M., Güneş K. and Gürses M. S., in Dyes and Pigments, Springer International Publishing, Cham, 2016, pp. 31–45, 10.1007/978-3-319-33892-7_3

[cit3] Waring D. R. and Hallas G., The Chemistry and Application of Dyes, Springer Science & Business Media, 2013

[cit4] Wang M., Funabiki K., Matsui M. (2003). Dyes Pigm..

[cit5] Benkhaya S., El Harfi S., El Harfi A. (2017). Appl. J. Environ. Eng. Sci..

[cit6] Ziarani G. M. , Moradi R., Lashgari N. and Kruger H. G., in Metal-Free Synthetic Organic Dyes, ed. G. M. Ziarani, R. Moradi, N. Lashgari and H. G. Kruger, Elsevier, 2018, pp. 47–93, 10.1016/B978-0-12-815647-6.00004-2

[cit7] Etesami H., Mansouri M., Habibi A., Jahantigh F. (2020). J. Mol. Struct..

[cit8] Khalik W. F., Ho L.-N., Ong S.-A., Wong Y.-S., Yusoff N. A., Lee S.-L. (2020). J. Environ. Health Sci. Eng..

[cit9] Pham T. D., Kobayashi M., Adachi Y. (2015). Colloid Polym. Sci..

[cit10] Gürses A. , Açıkyıldız M., Güneş K. and Gürses M. S., in Dyes and Pigments, Springer, 2016, pp. 13–29

[cit11] Erişkin S., Şener N., Yavuz S., Şener İ. (2014). Med. Chem. Res..

[cit12] Matada M. N., Jathi K., Rangappa M. M., Geoffry K., Kumar S. R., Nagarajappa R. B., Zahara F. N. (2020). J. King Saud Univ., Sci..

[cit13] Samad M. K., Hawaiz F. E. (2019). Bioorg. Chem..

[cit14] Tahir T., Ashfaq M., Saleem M., Rafiq M., Shahzad M. I., Kotwica-Mojzych K., Mojzych M. (2021). Molecules.

[cit15] Sahoo J., Mekap S. K., Kumar P. S. (2015). J. Taibah Univ. Sci..

[cit16] Kadhum A. A. H., Al-Amiery A. A., Musa A. Y., Mohamad A. B. (2011). Int. J. Mol. Sci..

[cit17] Aljamali N. M. (2015). Biochem. Anal. Biochem..

[cit18] MacDougall C. , in Goodman & Gilman's: The Pharmacological Basis of Therapeutics, 13e, ed. L. L. Brunton, R. Hilal-Dandan and B. C. Knollmann, McGraw-Hill Education, New York, NY, 2017

[cit19] Al-Rubaie L., Mhessn R. J. (2012). E-J. Chem..

[cit20] Pramanik P., Sahoo R., Das S. K., Halder M. (2020). Phys. Chem. Chem. Phys..

[cit21] Marchevsky E., Olsina R., Marone C. (1985). Talanta.

[cit22] Georgieva S., Bezfamilnyi A., Georgiev A., Varbanov M. (2021). Molecules.

[cit23] Bechtold T. and Pham T., in Textile Chemistry, De Gruyter, 2019, pp. 203–226, 10.1515/9783110549898-008

[cit24] Brown D., Laboureur P. (1983). Chemosphere.

[cit25] Matazo D. R., Ando R. A., Borin A. C., Santos P. S. (2008). J. Phys. Chem. A.

[cit26] Bureš F. (2014). RSC Adv..

[cit27] Del Nero J., De Araujo R., Gomes A., De Melo C. (2005). J. Chem. Phys..

[cit28] Çanakçı D. (2019). J. Mol. Struct..

[cit29] Nath I., Chakraborty J., Abednatanzi S., Van Der Voort P. (2021). Catalysts.

[cit30] Neifar M., Jaouani A., Kamoun A., Ellouze-Ghorbel R., Ellouze-Chaabouni S. (2011). Enzyme Res..

[cit31] Zhang J., Khayatnezhad M., Ghadimi N. (2022). Energy Sources, Part A.

[cit32] Han E., Ghadimi N. (2022). Sustain. Energy Technol. Assess..

[cit33] Bo G., Cheng P., Dezhi K., Xiping W., Chaodong L., Mingming G., Ghadimi N. (2022). Energy Sources, Part A.

[cit34] Wagner-Wysiecka E., Łukasik N., Biernat J. F., Luboch E. (2018). J. Inclusion Phenom. Macrocyclic Chem..

[cit35] Purwono B., Anwar C., Hanapi A. (2013). Indones. J. Chem..

[cit36] Patel A. R., Patel G., Maity G., Patel S. P., Bhattacharya S., Putta A., Banerjee S. (2020). ACS Omega.

[cit37] Al-Jorani K. R., Abbas R. F., Waheb A. A. (2021). Mater. Today: Proc..

[cit38] Al-Majidi S. M., Al-Khuzaie M. G. (2019). Iraqi J. Sci..

[cit39] Snigur D., Chebotarev A., Bevziuk K. (2018). J. Appl. Spectrosc..

[cit40] Kofie W., Amengor C., Orman E. (2016). Int. Res. J. Pure Appl. Chem..

[cit41] AlBaheley N. A., Fahad T. A., Ali A. A. (2021). J. Phys.: Conf. Ser..

[cit42] Cooper S. (1941). Ind. Eng. Chem., Anal. Ed..

[cit43] Cohen A. (1922). J. Am. Chem. Soc..

[cit44] Johnson A. H., Green J. R. (1930). Ind. Eng. Chem., Anal. Ed..

[cit45] Galenko E. E., Galenko A. V., Khlebnikov A. F., Novikov M. S., Shakirova J. R. (2016). J. Org. Chem..

[cit46] Adeniyi A. A., Ngake T. L., Conradie J. (2020). Electroanalysis.

[cit47] Zhou J., Wan X., Liu Y., Zuo Y., Li Z., He G., Long G., Ni W., Li C., Su X. (2012). J. Am. Chem. Soc..

[cit48] Gilani A. G., Taghvaei V., Rufchahi E. M., Mirzaei M. (2019). J. Mol. Liq..

[cit49] Hofmann D., Hofmann J., Hofmann L.-E., Hofmann L., Heinrich M. R. (2015). Org. Process Res. Dev..

[cit50] Liang M., Wang Z.-Y., Zhang L., Han H.-Y., Sun Z., Xue S. (2011). Renewable Energy.

[cit51] Becke A. D. (1992). J. Chem. Phys..

[cit52] Badal M. M. R., Ashekul Islam H. M., Maniruzzaman M., Abu Yousuf M. (2020). ACS omega.

[cit53] Azuki M., Morihashi K., Watanabe T., Takahashi O., Kikuchi O. (2001). J. Mol. Struct.: THEOCHEM.

[cit54] Tawarah K. M., Abu-Shamleh H. M. (1991). Dyes Pigm..

[cit55] Sanchez A. M., Barra M., de Rossi R. H. (1999). J. Org. Chem..

[cit56] Fan J., Shen X., Wang J. (1998). Anal. Chim. Acta.

[cit57] Abugri D. A., Apea O. B., Pritchett G. (2012). Green Sustainable Chem..

[cit58] Meeker E. W., Wagner E. (1933). Ind. Eng. Chem., Anal. Ed..

[cit59] Hong S. U., Singh S. P., Pyo M., Park W. B., Sohn K. S. (2017). Phys. Chem. Chem. Phys..

