# Supplementary material for: Synthesis, characterization and application of a novel polyazo dye as a universal acid–base indicator
Source: RSC Adv. 2022 Oct 3;12(43):28034–42. doi: 10.1039/d2ra04930a (PMC9527568; doi:10.1039/d2ra04930a)
Supplement: RA-012-D2RA04930A-s001 [file RA-012-D2RA04930A-s001.pdf]

Electronic Supplementary Material (ESI) for RSC Advances.
This journal is © The Royal Society of Chemistry 2022

Supporting Information

Synthesis, characterization and application of a novel polyazo dye as a universal acid-base indicator

Jannatul Naime,[a] *Muhammad Shamim Al Mamun,** [a] *Mohamed Aly Saad Aly,*[b] *Md. Maniruzzaman,*[c] *Md Mizanur Rahman Badal,*[c] *Kaykobad Md Rezaul Karim* [a]

[a]Chemistry Discipline, School of Science, Engineering and Technology, Khulna University, Khulna-9208, Bangladesh.

[b]Department of New Biology, Daegu Gyeongbuk Institute of Science and Technology (DGIST), 333 Techno jungang-daero, Daegu 42988, Republic of Korea

[c]Department of Chemistry, Khulna University of Engineering and Technology, Khulna-9203, Bangladesh

Table of Content

1. General Information……………………………………………………………..S2

2. Experimental……………………………………………………………….S2-S14

2.1 General Procedure of Reactant A………………………………………….....S2

2.2 General Procedure of Reactant B……………………………………………..S3

2.3 Investigation of Dissociation Constant………………………………………..S4-S5

2.4 Comparison with Methyl Orange……………………………………………..S5-S14

2.4.1 Titration Between Different Strength of Acids and Bases…………………..S6-S10

2.4.2 UV- Visible spectral Analysis during Different Types of Titrations……….S11-S14

3. Spectral Data……………………………………………………………...S14-S19

General Information:

All reagents were of reagent grade quality, where aniline ($C_6H_5NH_2$), hydrochloric acid (HCl) and sodium hydroxide (NaOH) were purchased from E. Merck (Germany), as well as sodium nitrite ($NaNO_2$) and sodium chloride (NaCl) were purchased from Sigma-Aldrich (Germany), and used without further purification. Thin Layer Chromatography (TLC) analyses were performed using Merck silica gel aluminum plates 60 F_{254}. SHIMADZU 4200 FT-IR spectrophotometer was used for recording IR-Spectra. ^{1}H-NMR spectra were recorded on BRUKER DPX 400 or DMX 500 instruments; chemical shifts are given in ppm and are relative

to Me_4Si as an internal standard or to the residual solvent peak on a Varian 500 Mz spectrometer AV-500 at 25°C. The absorption spectra of the compounds were scanned on UV-Visible double-beam spectrophotometer (LABOMED, INC, UVD-3200 PC) at 298 K through a quartz cell with a path length of 1 cm.

Experimental:

General Procedure of *p*- aminoazobenzene (PAAB)[11]

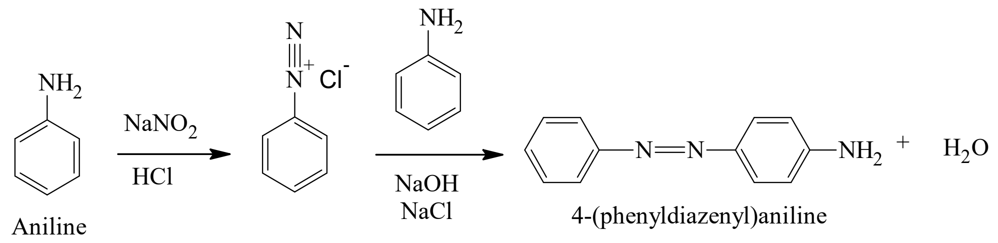

Scheme S1. Synthesis routes of *p*- aminoazobenzene (PAAB).

A solution of diazoaminobenzene (8 g, 40 mmol), aniline (17 mL, 180 mmol) and aniline hydrochloride (4 g, 30 mmol), was heated with the addition of $NaNO_2$ (4.2 mL, 95%) at 40 °C for 1 hour under continuous stirring. Next, glacial acetic acid (6 mL, 99%) was poured into the mixture and retained for 10 minutes. Following, crude product was dried, recrystallized with

CCl_4. The IR (KBr) spectrum of the product (ν cm^{-1}): 2375, 1475, 1400, 630. The synthesis procedure of PAAB is presented in **Scheme S1**.

General Procedure of Sodium 4-[(*E*)-{4-[(4-aminophenyl)diazenyl]phenyl} diazenyl]benzene sulfonate (SAPD$_2$BS) [1, 2]

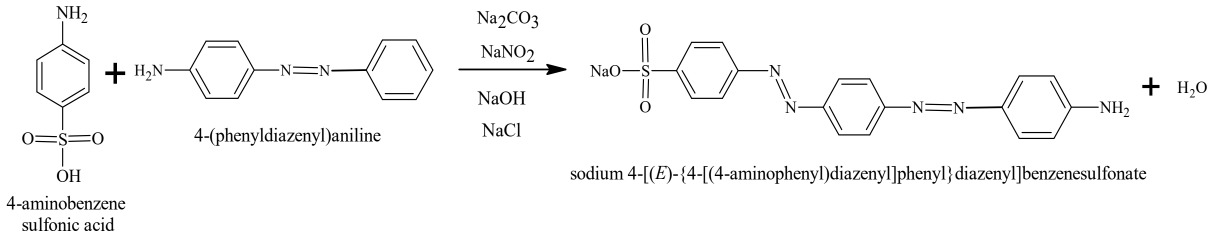

Scheme S2. Synthesis routes of Sodium 4-[(E)-{4-[(4-aminophenyl)diazenyl]phenyl} diazenyl]benzene sulfonate (SAPD$_2$BS).

A solution of sulfanilic acid (3 g, 17.34 mmol), sodium carbonate (Na_2CO_3, 7.5 mmol, 0.8 g) and 15 mL of water was first mixed in a round bottom flask. Following, the flask was placed in a hot (90 °C) water bath until a clear solution is obtained. Next sodium nitrite (1 g, 14.5 mmol, 98%) was added and then the mixture containing flask was placed in an ice water bath to obtain a significant amount of solid precipitate. Furthermore, a basic solution of PAAB (7 g, 35.6 mmol) was prepared as presented in **Scheme S2** of the SI, added into the above-mentioned mixture under continues stirring. Next, the flask containing the mixture was placed in an ice-water bath for 15 to 20 minutes. Following, the mixture was heated with sodium chloride (3 g) until dissolved, and then slowly cooled in an ice water bath for 30-40 minutes. Finally, the crude product was recrystallized with n-hexane and Dichloromethane (DCM), as shown in **Scheme S2**. Red powder, mp 178 ℃, yield 85%. The IR (KBr) spectrum of the product (ν cm^{-1}): 3041, 3381, 1654, 1600, 1400, 1371, 1170. ^{1}H NMR (400 MHz, $CDCl_3$): δ 8.06 (d, J=8.8 Hz, 2H, Ar), 7.92 (d, J=7.2 Hz, 2H, Ar), 7.56-7.51 (t, J=9.6 Hz, 4H, Ar), 7.59 (d, J=6.8 Hz, 2H, Ar), 7.54 (d, J=6.8 Hz, 2H, NH_2), 4.89 and 3.33 (MeOD).

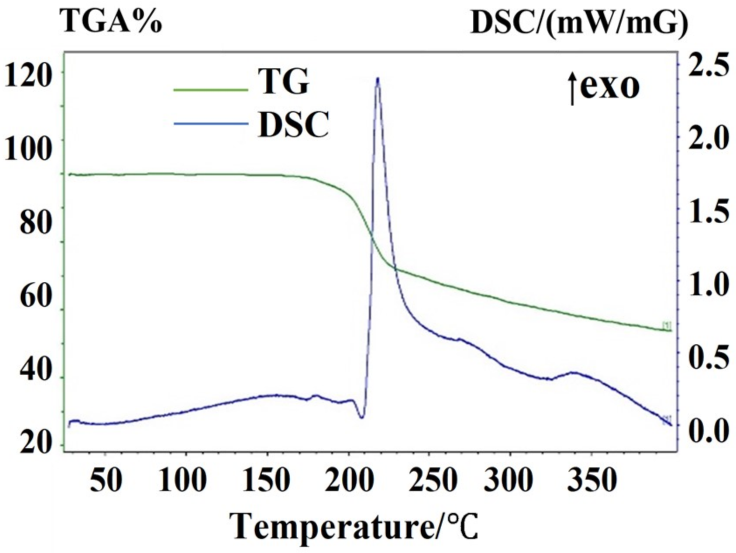

Figure S1. Thermo gravimetric analysis (TGA) and Differential scanning calorimetry (DSC) of $SDMAPD_3BS$.

Investigation of Dissociation Constant [33 44]

The buffer solution of different pH was prepared by mixing with both sodium hydroxide (NaOH) and boric acid. The synthesized compound ($SDMAPD_3BS$) was used as an indicator and the recorded absorbance at λ_{max} 387 nm and 454 nm was determined in the mixed aqueous solution of 10 wt% ethanol. The acidic form of $SDMAPD_3BS$ (exists alone in acidic solutions) absorbed light in the green spectral range at a wavelength of $\lambda_{max} \approx$ 455nm, and appeared as the complementary color red. On the other hand, the yellow color form existing alone in basic solutions absorbed light in the violet spectral range at a wavelength of $\lambda_{max} \approx$ 394 nm. The absorbance and dissociation constant (K_d) values of $SDMAPD_3BS$ are recorded in **Table S1** as well as the linear relationship between pK and pH has shown in **Figure S2.**

Table S1. Absorbance and Dissociation Constant K_d of $SDMAPD_3BS$.

Volume of 0.2M NaOH (mL)	Volume of Boric Acid 0.2M(mL)	pH of the Solution	Absorbance (A)
0.0	10	1.7	0.898
0.5	9.5	2.5	0.892
1.0	9.0	4.4	0.887
1.5	8.5	5.3	0.882
2.0	8.0	6.1	0.878
2.5	7.5	7.0	0.873
3.0	7.0	8.1	0.868
3.5	6.5	8.8	0.861
4.0	6.0	9.1	0.859
4.5	5.5	9.5	0.853
5.0	5.0	9.8	0.847
5.5	5.5	10.2	0.842
Na_2CO_3	0.0	10.5	0.864

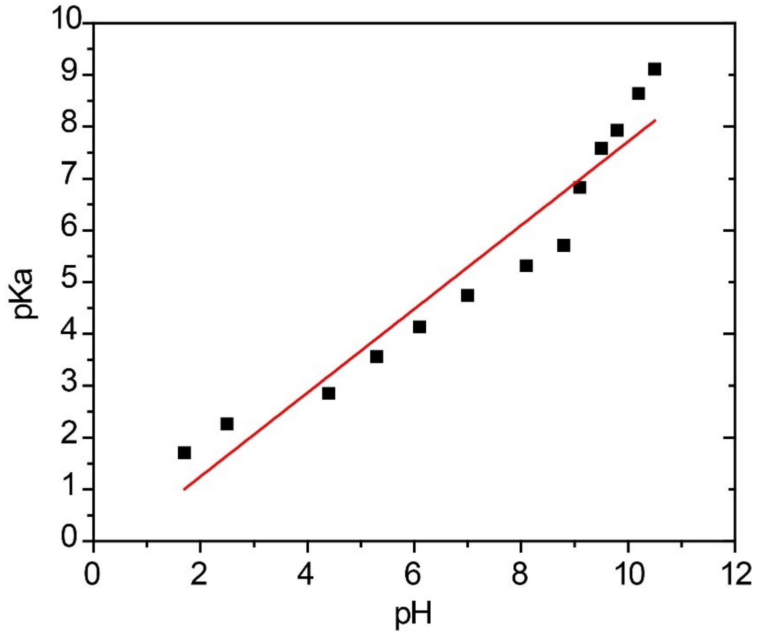

Figure S2. The comparison of dissociation constant between $SDMAPD_3BS$ and MO.

Comparison with Methyl Orange:

$SDMAPD_3BS$ showed four different colors (blackish red, reddish orange, orange and yellow) at different pH ranges within the pH range of 1-14, as summarized in **Table 2S**. However, among these colors, there were three prominent colors observed in a perfect pH gap, indicating the acidic and basic medium of any solution along with providing the exact endpoint.

pH	Compound Name	
	**Methyl Orange**	**Synthesized Compound**
**1**	Red	Blackish Red
**2**	Red	Blackish Red
**3**	Red	Reddish Orange
**4**	Reddish Orange	Reddish Orange
**5**	Yellowish Orange	Orange
**6**	Yellow	Orange
**7**	Yellow	Orange
**8**	Yellow	Light Orange
**9**	Yellow	Yellow Orange
**10**	Yellow	Yellow
**11**	Yellow	Yellow
**12**	Yellow	Yellow
**13**	Faint Yellow	Yellow
**14**	Faint Yellow	Yellow

Table S2. Change of color with pH variation.

Titration between different strength of acids and bases:

Titration of the acidic solution was performed with 2-3 drops of both indicators (Methyl orange and synthesized compound) in two different conical flasks, pH meter was placed into the conical flask, and the base was added through a burette drop wise. The color of the solution was dramatically changed when 1-2 drops of either indicator was added, as shown in **Figure S3**. Furthermore, the endpoints for those titrations were recorded and presented in **Table S3 - Table S9**.

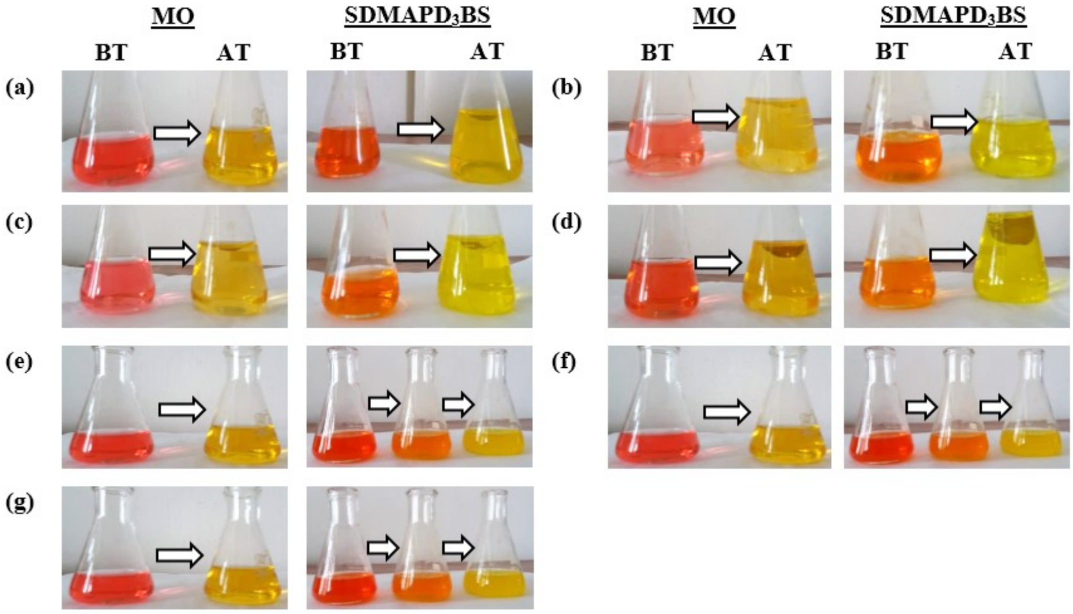

Figure S3. Titration between strong acid and strong base with the end points for MO and $SDMAPD_3BS$ are 10.7 and 11.6, respectively, (a); weak acid and strong base with the end points for MO and $SDMAPD_3BS$ are 10.4 and 11.5, respectively, (b); strong acid and weak base with the end points for MO and $SDMAPD_3BS$ are 6.1 and 6.7, respectively, (c); weak acid and weak base with the end points for MO and $SDMAPD_3BS$ are 7.9 and 9.1, respectively, (d); ploybasic acid and strong base with the end points for MO and $SDMAPD_3BS$ are 10.7 and (7.5, 11.6), respectively, (e); strong acid + weak acid and strong base with the end points for MO and $SDMAPD_3BS$ are 10.6 and (7.4, 11.6), respectively, (f); and strong acid + weak acid and strong base + weak base with the end points for MO and $SDMAPD_3BS$ are 9.7 and (3.8, 9.9), respectively, (g).

*MO: Methyl Orange
*$SDMAPD_3BS$: Synthesized Compound
*BT: Before Titration
*AT: After Titration

Table S3. Titration between Strong Acid and Strong Base.

NaOH Added (mL)	HCl Unreacted (mL)	% of Reaction	Total Volume of Solution (mL)	pH	
				MO	$SDMAPD_3BS$
0.0	10.0	0.0	10.0	0.8	0.7
1.0	9.0	10.0	11.0	0.9	0.8
2.0	8.0	20.0	12.0	1.0	0.9
3.0	7.0	30.0	13.0	1.1	1.0
4.0	6.0	40.0	14.0	1.2	1.1
5.0	5.0	50.0	15.0	1.3	1.2
6.0	4.0	60.0	16.0	1.5	1.3
7.0	3.0	70.0	17.0	1.7	1.4
8.0	2.0	80.0	18.0	1.9	1.5
9.0	1.0	90.0	19.0	2.2	1.6
9.5	0.5	95.0	19.5	2.5	1.7
9.8	0.2	98.0	19.8	2.9	1.9
**9.9**	**0.1**	**99.0**	**19.9**	**3.4**	**2.4**
**10.0**	**0.0**	**100.0**	**20.0**	**10.6**	**11.7**
10.1	0.0	-	20.1	11.5	12.1
10.5	0.0	-	20.5	11.8	12.4
11.0	0.0	-	21.0	12.0	12.7
11.5	0.0	-	21.5	12.1	12.9
12.0	0.0	-	22.0	12.2	13.1
12.5	0.0	-	22.5	12.3	13.2
13.0	0.0	-	23.0	12.3	13.2

Table S4. Titration between Weak Acid and Strong Base.

NaOH Added (mL)	CH_3COOH Unreacted (mL)	% of Reaction	Total Volume of Solution (mL)	pH	
				MO	$SDMAPD_3BS$
0.0	10.00	0.0	10.0	2.5	2.5
1	1.25	87.50	11.0	3.1	3.5
2	0.63	93.70	12.0	3.3	3.9
3	0.32	96.80	13.0	3.5	4.2
3.5	0.13	98.70	13.5	3.8	4.4
4	0.08	99.20	14.0	3.9	4.6
4.5	0.05	99.50	14.5	4.1	4.8
**4.7**	**0.0063**	**99.93**	**14.7**	**4.8**	**5.3**
**4.9**	**0.0**	**99.99**	**14.9**	**10.4**	**11.5**
5.3	0.0	-	15.3	10.9	12.1
5.4	0.0	-	15.4	11.1	12.3
5.5	0.0	-	15.5	11.3	12.4

Table S5. Titration between Strong Acid and Weak Base.

NH_4OH Added (mL)	HCl Unreacted (mL)	% of Reaction	Total Volume of Solution (mL)	pH	
				MO	$SDMAPD_3BS$
0.0	10.0	0.0	10.0	1	0.7
3.0	7.9	21.0	13.0	1.1	0.8
6.0	6.3	37.0	16.0	1.2	0.9
9.0	5.0	50.0	19.0	1.3	1
12.0	4.0	60.0	22.0	1.4	1.1
14.0	3.2	68.0	24.0	1.5	1.2
16.0	2.5	75.0	26.0	1.6	1.3
18.0	2.0	80.0	28.0	1.7	1.5
19.0	1.6	84.0	29.0	1.8	1.7
19.5	1.0	90.0	29.5	2.1	1.9
19.9	**0.63**	**93.7**	**29.9**	**2.9**	**2.4**
**20.1**	**0.0002**	**99.9**	**30.1**	**6.1**	**6.7**
21.0	0.00002	-	31.0	6.7	7.5
22.0	0.0	-	32.0	7.4	7.9
23.0	0.0	-	33.0	7.8	8.2
24.0	0.0		34.0	8.2	8.5
25.0	0.0	-	35.0	8.5	8.7
26.0	0.0	-	36.0	8.8	8.9
28.0	0.0	-	38.0	9.1	9.1
30.0	0.0	-	40.0	9.5	9.5
32.0	0.0	-	42.0	10.2	10
34.0	0.0	-	44.0	11.6	11.5

Table S6. Titration between Weak Acid and Weak Base.

NH_4OH Added (mL)	CH_3COOH Unreacted (mL)	% of Reaction	Total Volume of Solution (mL)	pH	
				MO	$SDMAPD_3BS$
0.0	10.0	0.0	10.0	2.5	2.5
1.0	9.0	10.0	11.0	3.7	3.5
3.0	7.0	30.0	13.0	4.2	3.9
5.0	5.0	50.0	15.0	4.5	4.1
7.0	3.0	70.0	17.0	4.7	4.3
9.0	1.0	90.0	19.0	4.9	4.5
9.5	0.5	95.0	19.5	5.1	4.6
**9.8**	**0.2**	**98.0**	**19.8**	**5.5**	**5.3**
**10.0**	**0.0**	**100.0**	**20.0**	**7.9**	**9.1**
11.0	0.0	-	21.0	8.4	9.4
13.0	0.0	-	23.0	8.7	9.6
15.0	0.0	-	25.0	8.9	9.7
17.0	0.0	-	27.0	9.1	9.8

Table S7. Titration between Polybasic Acid and Strong Base.

NaOH Added (mL)	H_2SO_4 Unreacted (mL)	% of Reaction	Total Volume of Solution (mL)	pH	
				MO	SDMAPD$_3$BS
0.0	10.0	0.0	10.0	0.2	0.2
1.0	8.78	12.2	11.0	0.4	0.4
2.0	7.56	24.4	12.0	0.8	0.8
3.0	6.34	36.6	13.0	1.2	1.2
3.5	5.12	48.8	13.5	1.5	1.6
4.0	3.90	61.0	14.0	1.7	1.8
4.5	3.29	67.1	14.5	2.0	2.0
**4.8**	**2.92**	**70.8**	**14.8**	2.5	**2.3**
**4.9**	**2.80**	**72.0**	**14.9**	**4.1**	**7.5**
**5.0**	**2.68**	**73.2**	**15.0**	**10.7**	7.6
5.5	2.07	79.3	15.5	11.3	7.8
6.0	1.46	85.4	16.0	11.4	7.9
6.7	0.61	93.9	16.7	11.5	8.1
**7.1**	**0.12**	**98.8**	**17.1**	11.6	**8.4**
**7.2**	**0.00**	**100.0**	**17.2**	11.7	**11.6**
7.5	-	-	17.5	11.8	12.1
7.9	-	-	17.9	11.9	12.5
8.2	-	-	18.2	12.0	12.8
8.5	-	-	18.5	12.1	13.0

Table S8. Titration between Strong Acid + Weak Acid and Strong Base.

NaOH Added (mL)	Boric Acid+HCl Unreacted (mL)	% of Reaction	Total Volume of Solution (mL)	pH	
				MO	SDMAPD$_3$BS
0.0	10.0	0.0	10.0	0.1	0.1
1.0	8.57	14.3	11.0	0.3	0.3
2.0	7.14	28.6	12.0	0.5	0.5
2.5	6.42	35.8	12.5	0.7	0.7
**3.0**	**5.71**	**42.9**	**13.0**	0.8	1.4
**3.1**	**5.57**	**44.3**	**13.1**	0.9	**2.1**
3.4	5.14	48.6	13.4	1.1	**7.4**
3.8	4.57	54.3	13.8	1.5	7.7
4.2	4.00	60.0	14.2	2.1	7.8
**4.6**	**3.71**	**62.9**	**14.6**	2.5	8.0
**4.9**	**3.57**	**64.3**	**14.9**	**3.2**	**8.2**
**5.1**	**3.29**	**67.1**	**15.1**	**10.6**	**11.6**
5.5	2.86	71.4	15.5	11.1	11.9
6.0	1.42	85.8	16.0	11.5	12.1
7.0	0.00	100.0	17.0	11.8	12.2

Table S9. Titration between Strong Acid + Weak Acid and Strong Base + Weak Base

NaOH + NH_4OH Added (mL)	Boric Acid + HCl Unreacted (mL)	% of Reaction	Total Volume of Solution (mL)	pH	
				MO	$SDMAPD_3BS$
0.0	10.0	0.0	10.0	0.5	0.7
2.0	8.77	12.3	12.0	0.6	1.2
4.0	7.55	24.5	14.0	0.8	1.7
**4.5**	**7.24**	**27.6**	**14.5**	0.9	**2.2**
**4.7**	**7.12**	**28.8**	**14.7**	0.9	**3.8**
5.0	6.93	30.7	15.0	1.0	4.3
6.0	5.70	4.30	16.0	1.3	4.7
7.0	3.86	61.4	17.0	1.7	5.3
8.0	2.02	79.8	18.0	2.2	5.9
9.0	0.80	92.00	19.0	2.9	6.4
**9.5**	**0.18**	**98.2**	**19.5**	**3.8**	**6.8**
**9.9**	**0.00**	**100.00**	**19.9**	**9.7**	**9.9**
10.5	-	-	20.5	10.2	10.3
11.0	-	-	21.0	10.6	10.7
12.0	-	-	22.0	10.9	10.9
13.0	-	-	23.0	11.1	11.1
14.0	-	-	24.0	11.3	11.3
15.0	-	-	25.0	11.4	11.5

UV- Visible Spectral Analysis during Different Types of Titrations

During titration, the pH of the solution was changed from lower pH to higher pH and the absorbance showed either hypsochromic shift or blue shift. In titration between a strong acid and strong base: the equivalence point (EP) was 7.0 for MO and 7.05 for $SDMAPD_3BS$, the inflection points (IP) were 3.4 and 10.7 for MO and 2.4 and 11.6 for $SDMAPD_3BS$, and the pH changing range for $SDMAPD_3BS$ (9.2) was greater than for MO (7.3). In titration between a weak acid and a strong base, EP was 7.6 for MO and 8.4 for $SDMAPD_3BS$, IP is 4.8 and 10.4 for MO and 5.3 and 11.5 for $SDMAPD_3BS$, and the pH changing range for $SDMAPD_3BS$ (6.2) is greater than for MO (5.6). In titration between a strong acid and weak base, EP is 4.5 for MO and 4.55 for $SDMAPD_3BS$, IP is 2.9 and 6.1 for MO and 2.4 and 6.7 for $SDMAPD_3BS$, and the pH changing range for $SDMAPD_3BS$ (4.3) is greater than for MO (3.2). In titration

between a weak acid and weak base, EP is 6.7 for MO and 7.2 for $SDMAPD_3BS$, IP is 5.5 and 7.9 for MO and 5.3 and 9.1 for $SDMAPD_3BS$, and the pH changing range for $SDMAPD_3BS$ (3.8) is greater than for MO (2.4), as shown in **Figure S4**. In titration between polybasic acid and strong base, EP is 7.4 for MO and two values of (4.9, 10.0) for $SDMAPD_3BS$, IP is 4.1 and 10.7 for MO and two sets of (2.3, 7.5) and (8.4, 11.6) for $SDMAPD_3BS$, and the pH changing range for $SDMAPD_3BS$ (3.2, 5.2) is less than for MO (6.6). In this case, $SDMAPD_3BS$ contains two pK values of 3.5 and 9.1. In titration between strong acid + weak acid and strong base, EP is 6.9 for MO and two values of (4.75, 9.90) for $SDMAPD_3BS$, IP is 3.2 and 10.6 for MO and two sets of (2.1, 7.4) and (8.2, 11.8) for $SDMAPD_3BS$, and the pH changing range for $SDMAPD_3BS$ (3.6, 5.3) is less than for MO (7.4). In this case, $SDMAPD_3BS$ contains two pK values of 3.3 and 9.2. In titration between strong acid + weak acid and strong base + weak base, EP is 6.87 for MO and two EP (3.0, 8.35) for $SDMAPD_3BS$, IP is 3.8 and 9.9 for MO and two sets of (2.2, 3.8) and (6.8, 9.9) for $SDMAPD_3BS$, and the pH changing range for $SDMAPD_3BS$ (1.6, 3.1) is less than for MO (6.1). In this case, $SDMAPD_3BS$ contains two pK values of 2.8 and 8.6, as show in **Figure S5**.

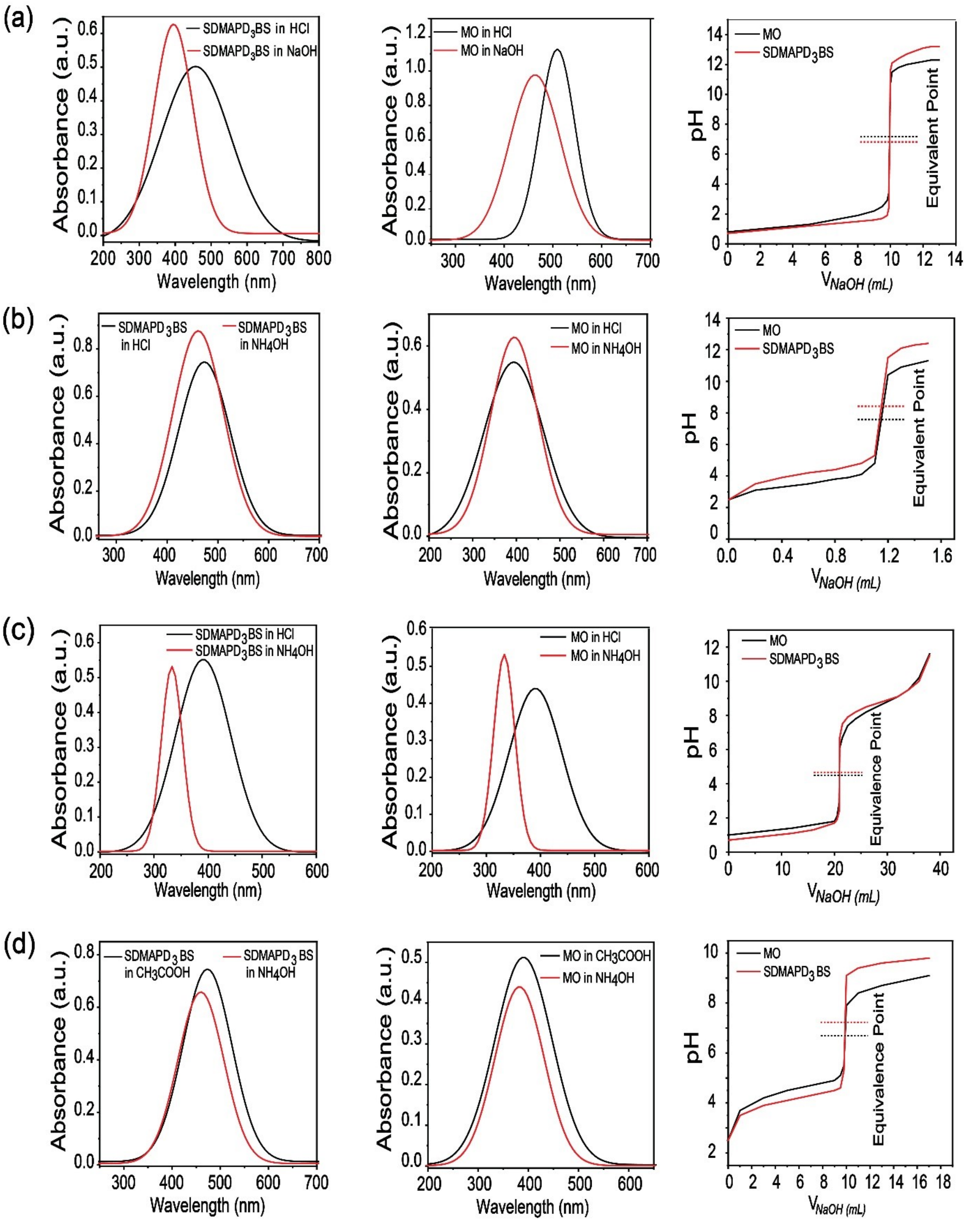

Figure S4. UV- Visible spectra of the acidic solution was shifted from right to left and titration between strong acid (HCl) and strong base (NaOH) (a), weak acid (CH_3COOH) and strong base (NaOH) (b), strong acid (HCl) and weak base (NH_4OH) (c), weak acid (CH_3COOH) and weak base (NH_4OH) (d).

*MO: Methyl Orange

* $SDMAPD_3BS$: Synthesized compound

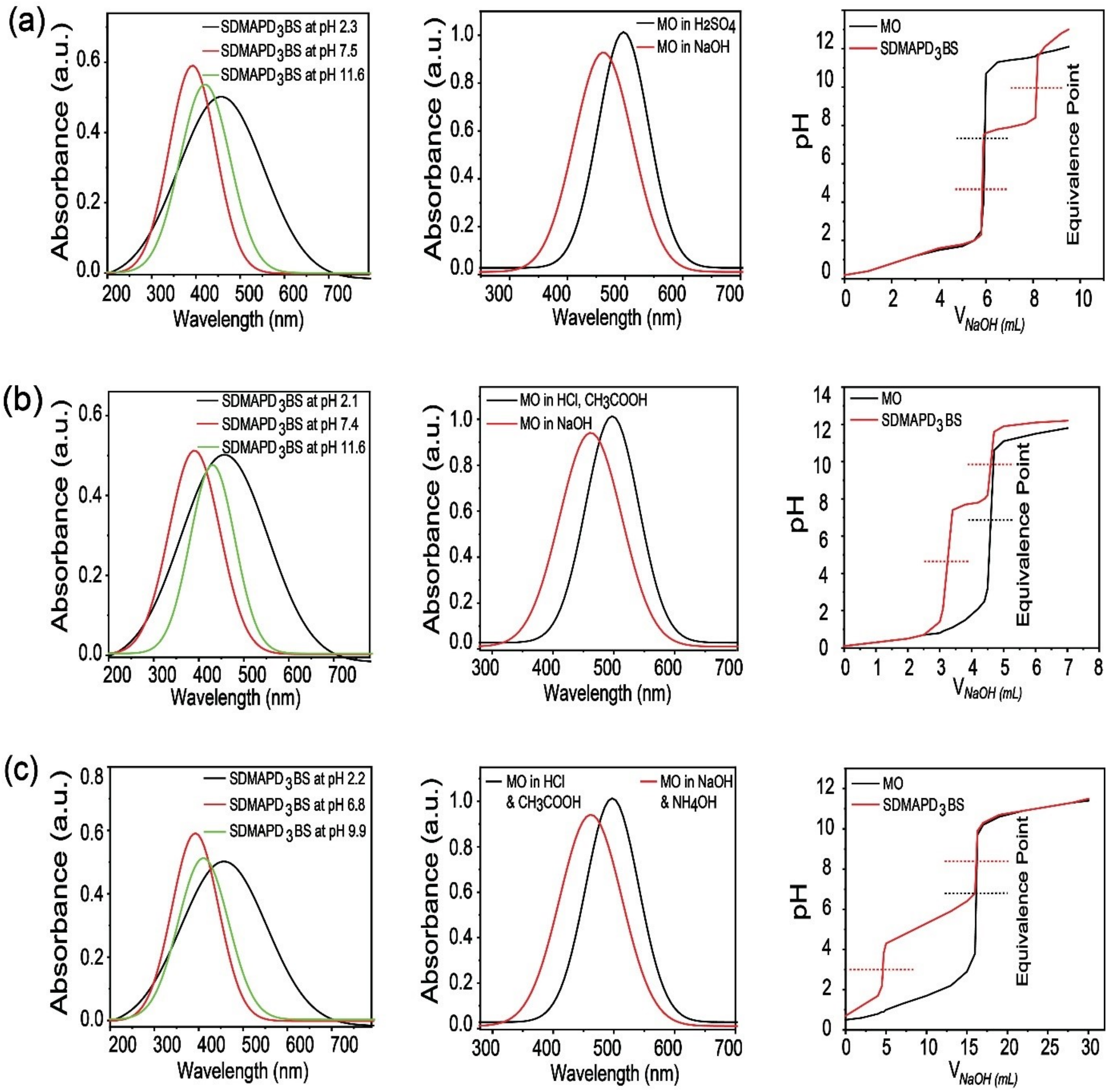

Figure S5. polybasic acid (H_2SO_4) and strong base (NaOH) (a), strong acid (HCl) + weak acid (CH_3COOH) and strong base (NaOH) (b), strong acid (HCl) + weak acid (CH_3COOH) and strong base (NaOH) + weak base (NH_4OH) (c).
*MO: Methyl Orange
* $SDMAPD_3BS$: Synthesized compound

The structure of $SDMAPD_3BS$ is mostly analogous to MO with some dissimilarities. It is noteworthy that increasing the number of azo groups can affect the solubility of the compound in polarity basis, and further increase the stability of the compound.[55] From the structural information, $SDMAPD_3BS$ has three azo groups while MO has one azo group, so, $SDMAPD_3BS$ is more stable and less dissociated compared with MO. Furthermore,

$SDMAPD_3BS$ have two pH ranges: one range (2.1 – 3.8) fits within strong acidic titration, while the other range (8.2 – 9.8) fits within weak basic titration. However, methyl orange has only one pH range (3.1 – 4.2). $SDMAPD_3BS$ exists in two pH ranges, therefore it forms equivalence point in same type of titrations unlike MO, as can be observed in **Figure S4** and **Figure S5**. $SDMAPD_3BS$ displayed color change in titration as an indicator similar to MO. However, MO has one azo group so it can take only one proton from media to convert from basic nature to acidic nature.[66] $SDMAPD_3BS$ has two pH ranges and three azo groups. At pH range of (8.2 – 9.8) it shows a yellow color when it takes one proton in first step similar to MO. Similarly, at pH range of (2.1 – 3.8), it displays an orange color when it takes two protons. Furthermore, SDMAPD3BS reveals different equivalence points in individual pH ranges (at different titrations). Additionally, $SDMAPD_3BS$ reveals two pK_a values in different types of titrations, as presented in **Figure S4 and Figure S5** and **Table S3-S9**.

Spectral Data:

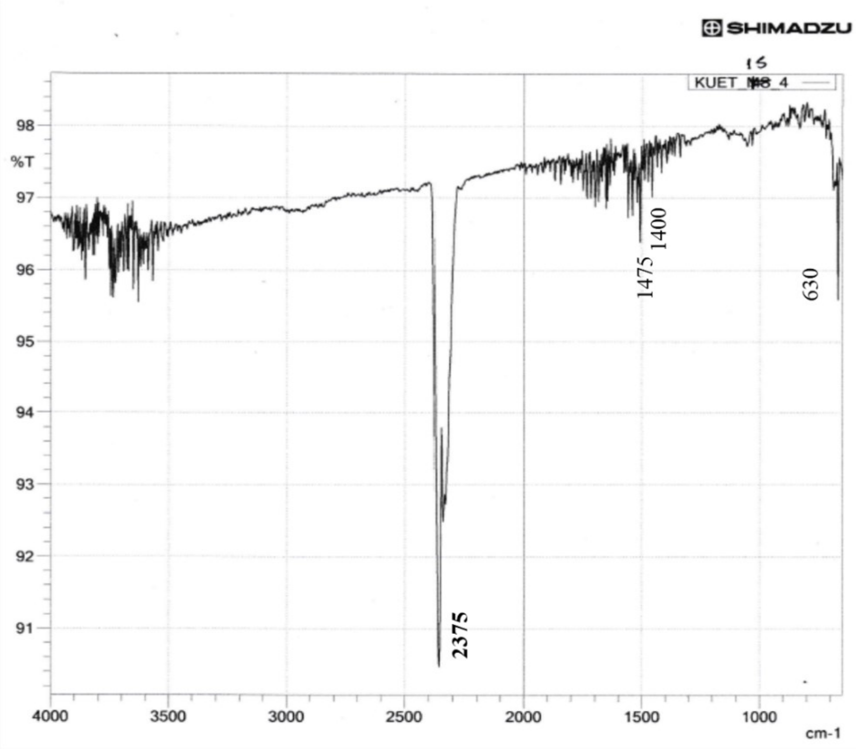

Figure S6. FT-IR Spectrum of PAAB.

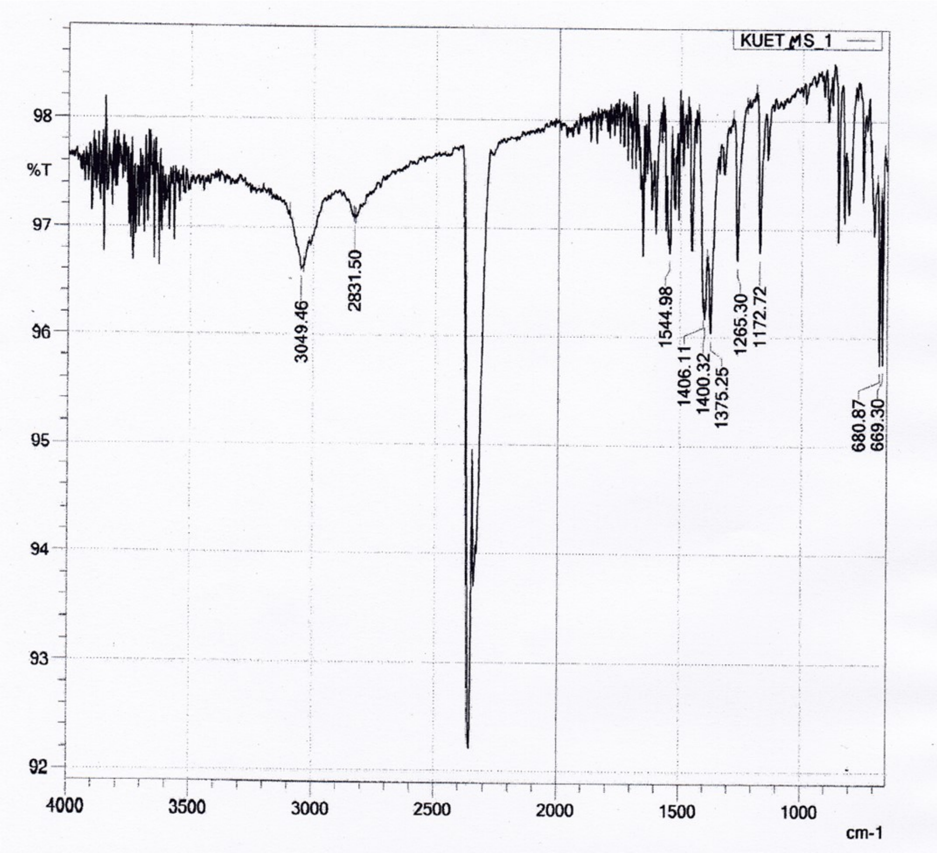

Figure S7. FT-IR Spectrum of $SAPD_2BS$.

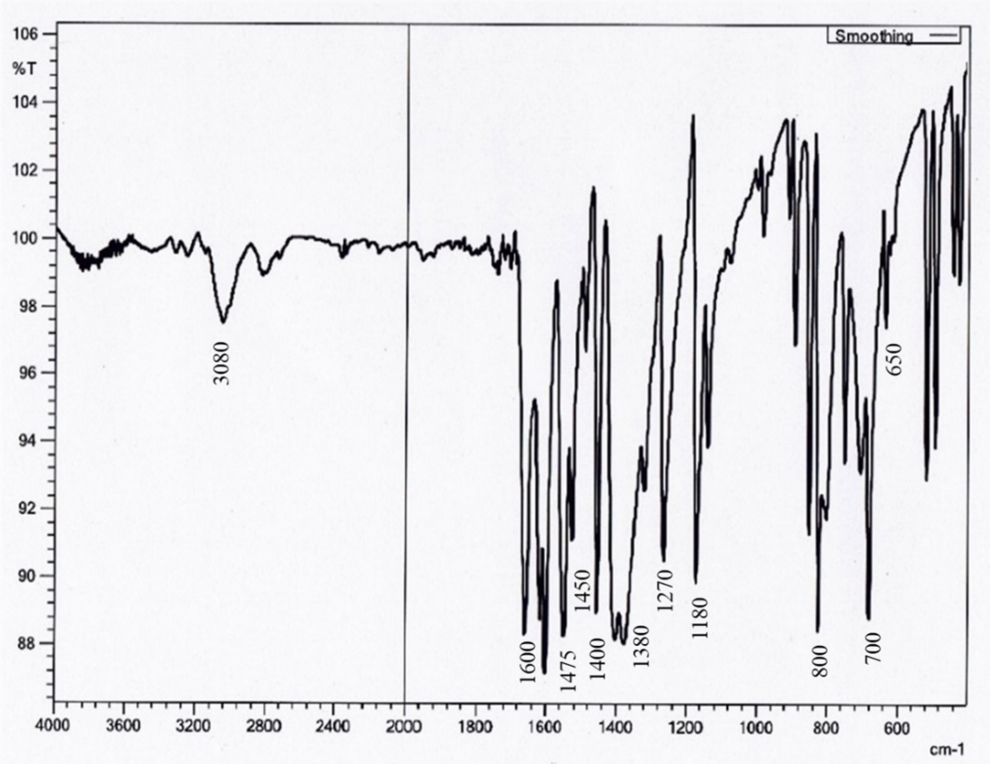

Figure S8. FT-IR spectrum of $SDMAPD_3BS$.

(a)

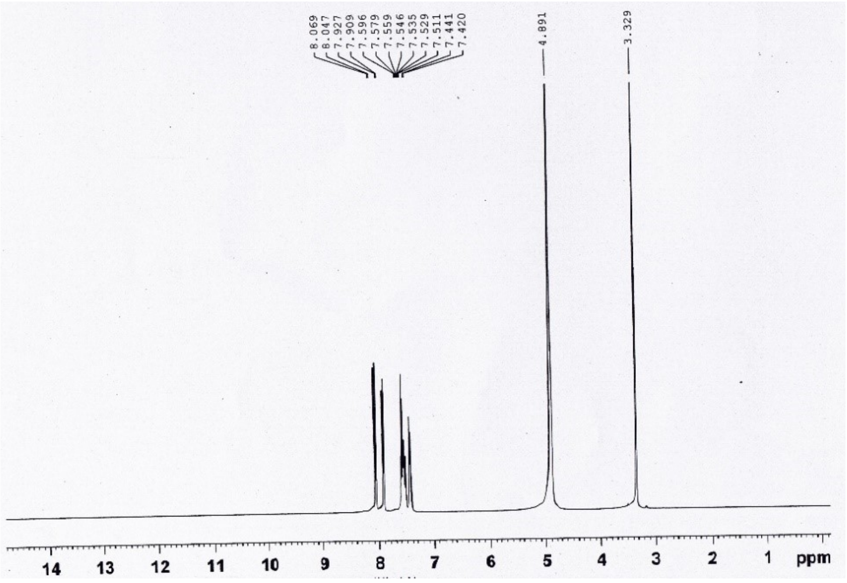

(b)

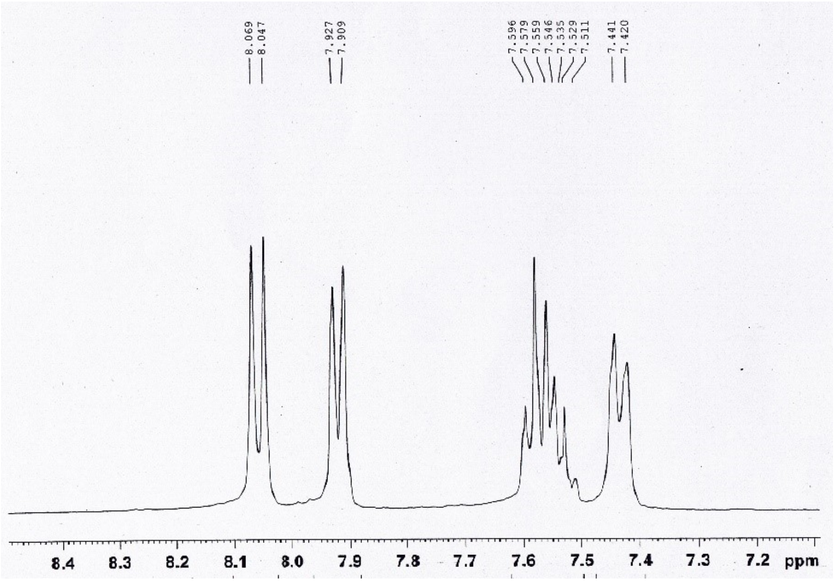

Figure S9. Compressed image (a) and expanded image (b) of the 1H-NMR spectrum of $SAPD_2BS$.

(a)

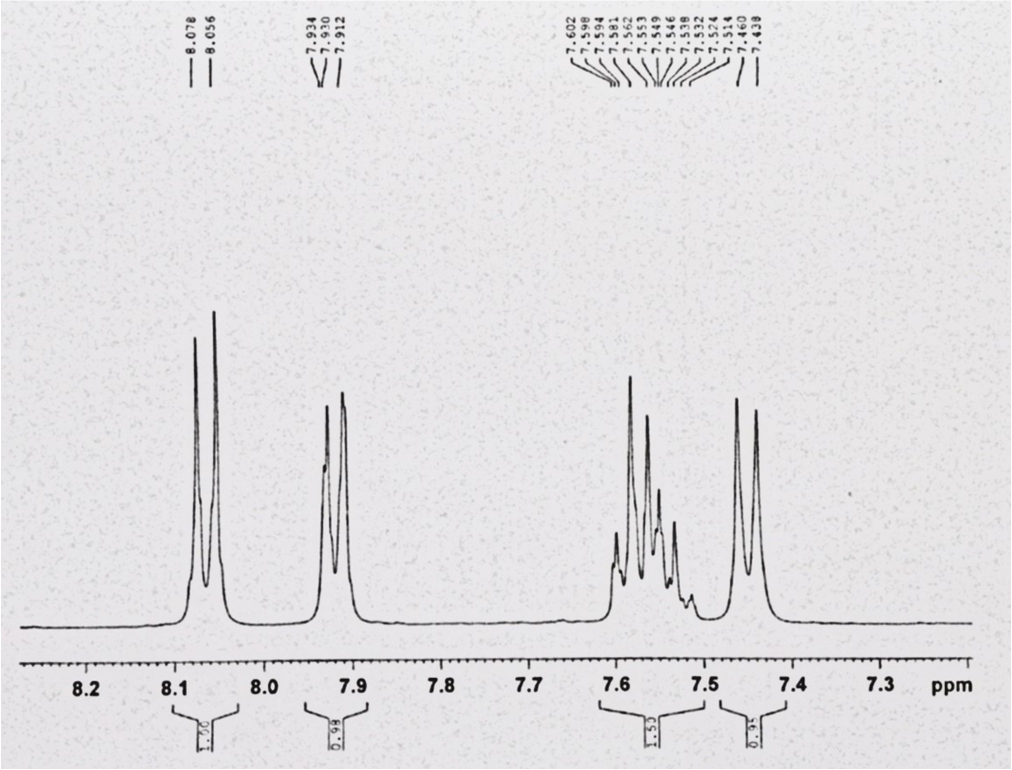

(b)

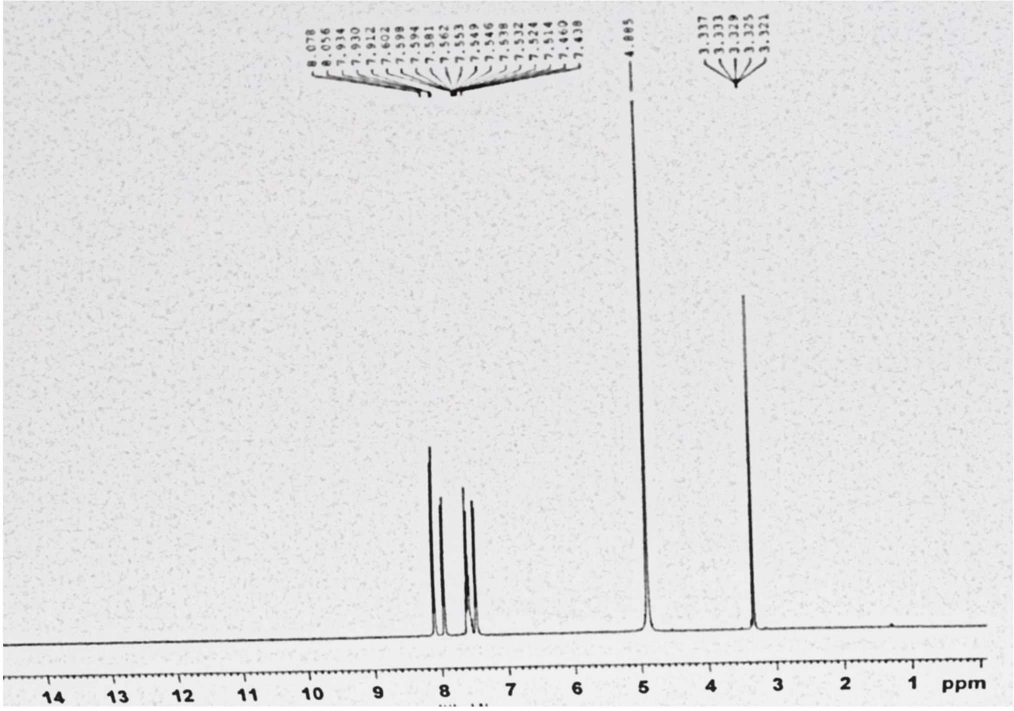

Figure S10. Expanded (a) and compressed (b) images of 1H-NMR spectrum of $SDMAPD_3BS$.

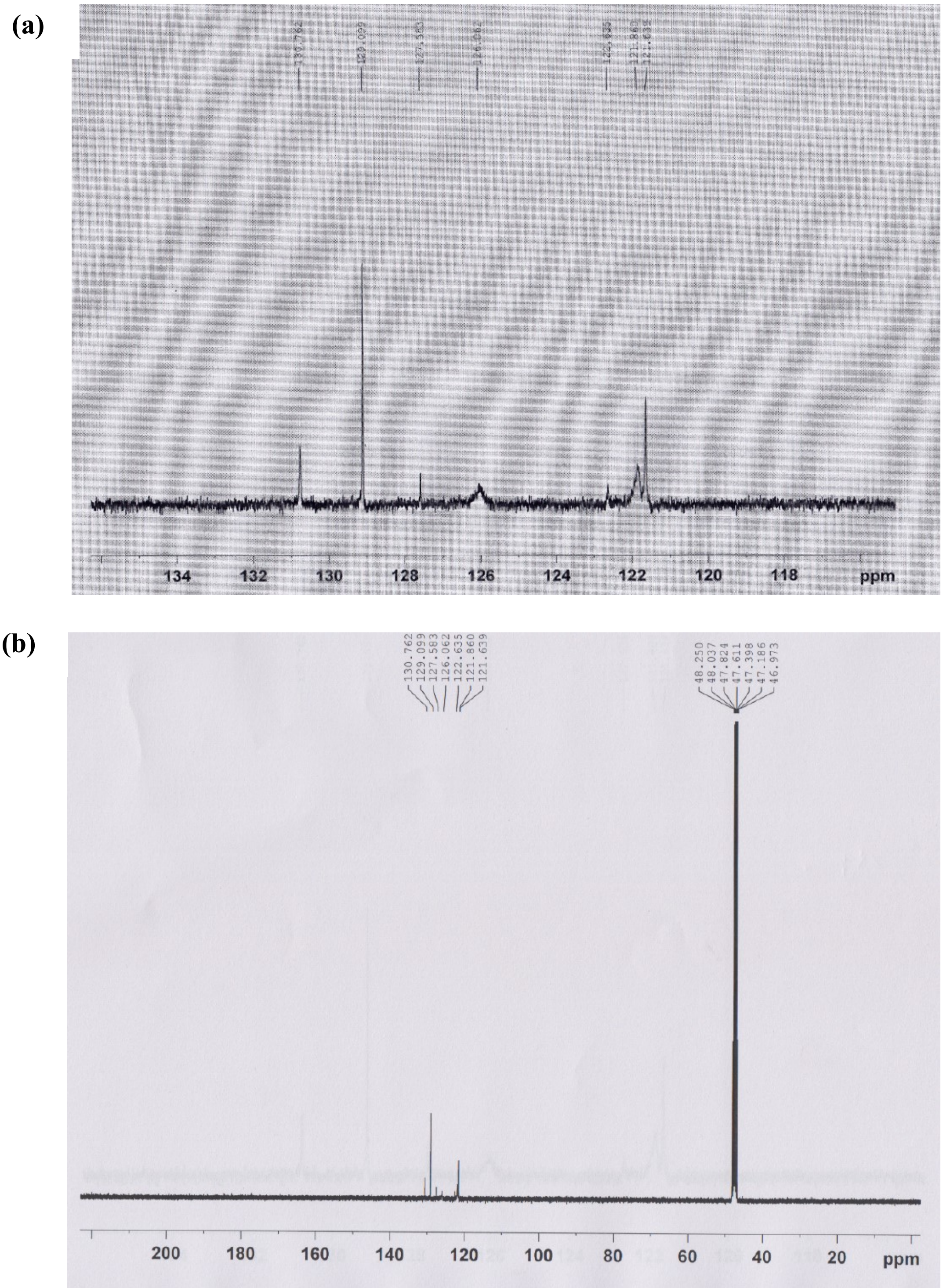

Figure S11. Expanded (a) and compressed (b) images of ^{13}C-NMR of $SDMAPD_3BS$.

References:

1. M. Liang, Z.-Y. Wang, L. Zhang, H.-Y. Han, Z. Sun and S. Xue, *Renewable Energy*, 2011, **36**, 2711-2716.
2. D. Hofmann, J. Hofmann, L.-E. Hofmann, L. Hofmann and M. R. Heinrich, *Organic Process Research & Development*, 2015, **19**, 2075-2084.
3. B. John, in *Advances in Inorganic Chemistry*, Elsevier, 2013, vol. 65, pp. 217-310.
4. B. Tanganov and I. Alekseeva, *Russian journal of general chemistry*, 2006, **76**, 1724-1728.
5. J. Del Nero, R. De Araujo, A. Gomes and C. De Melo, *The Journal of chemical physics*, 2005, **122**, 104506.
6. A. G. Gilani, V. Taghvaei, E. M. Rufchahi and M. Mirzaei, *Journal of Molecular Liquids*, 2019, **273**, 392-407.
